# Neuroregenerative potential of intravenous G-CSF and autologous peripheral blood stem cells in children with cerebral palsy: a randomized, double-blind, cross-over study

**DOI:** 10.1186/s12967-017-1120-0

**Published:** 2017-01-21

**Authors:** Wee-Jin Rah, Young-Ho Lee, Jin-Hwa Moon, Hyun-Ju Jun, Hye-Ryeong Kang, Hani Koh, Hye Jung Eom, Ji Young Lee, Young Jun Lee, Ji Young Kim, Yun-Young Choi, Kyeongil Park, Mi Jung Kim, Seung-Hyun Kim

**Affiliations:** 1 Department of Pediatrics, Hanyang University Medical Center, 222-1, Wangsimni-ro, Seongdong-gu, Seoul, 04763 South Korea; 2 Blood and Marrow Transplantation Center, Hanyang University Medical Center, Seoul, South Korea; 3 Cell Therapy Center, Hanyang University Medical Center, Seoul, South Korea; 4 Department of Radiology, Hanyang University Medical Center, Seoul, South Korea; 5 Department of Nuclear Medicine, Hanyang University Medical Center, Seoul, South Korea; 6 Department of Rehabilitation Medicine, Hanyang University Medical Center, Seoul, South Korea

**Keywords:** Cerebral palsy, Granulocyte colony-stimulating factor, Mobilized peripheral blood mononuclear cells, Neuroregeneration

## Abstract

**Objective:**

We performed a randomized, double-blind, cross-over study to assess the neuroregenerative potential of intravenous granulocyte colony-stimulating factor (G-CSF) followed by infusion of mobilized peripheral blood mononuclear cells (mPBMCs) in children with cerebral palsy (CP).

**Methods:**

Children with non-severe CP were enrolled in this study. G-CSF was administered for 5 days, then mPBMCs were collected by apheresis and cryopreserved. One month later (M1), recipients were randomized to receive either mPBMCs or a placebo infusion, and these treatment groups were switched at 7 months (M7) and observed for another 6 months (M13). We assessed the efficacy of treatment by evaluating neurodevelopmental tests, as well as by brain magnetic resonance imaging-diffusion tensor imaging (MRI-DTI) and ^18^F-fluorodeoxyglucose (FDG) brain positron emission tomography-computed tomography (PET-CT) scanning to evaluate the anatomical and functional changes in the brain.

**Results:**

Fifty-seven patients aged 4.3 ± 1.9 (range 2–10) years and weighing 16.6 ± 4.9 (range 11.6–56.0) kg were enrolled in this study. The administration of G-CSF as well as the collection and reinfusion of mPBMCs were safe and tolerable. The yield of mPBMCs was comparable to that reported in studies of pediatric donors without CP and patients with nonhematologic diseases. 42.6% of the patients responded to the treatment with higher neurodevelopmental scores than would normally be expected. In addition, larger changes in neurodevelopment test scores were observed in the 1 month after G-CSF administration (M0–M1) than during the 6 months after reinfusion with mPBMCs or placebo (M1–M7 or M7–M13). Patients who received G-CSF followed by mPBMC infusion at 7 months (T7 group) demonstrated significantly more neurodevelopmental improvement than patients who received G-CSF followed by mPBMC infusion at 1 month (T1 group). In contrast to the results of neurodevelopment tests, the results of MRI-DTI at the end of this study showed greater improvement in the T1 group. Although we observed metabolic changes to the cerebellum, thalamus and cerebral cortex in the ^18^F-FDG brain PET-CT scans, there were no significant differences in such changes between the mPBMC and placebo group or between the T1 and T7 group.

**Conclusions:**

Neurodevelopmental improvement was seen in response to intravenous G-CSF followed by mPBMC reinfusion, particularly to the G-CSF alone even without mPBMC reinfusion. Further studies using a larger number of mPBMCs for the infusion which could be collected by repeated cycles of apheresis or using repeated cycles of G-CSF alone, are needed to clarify the effect of mPBMC reinfusion or G-CSF alone (Trial registration: ClinicalTrials.gov, NCT02983708. Registered 5 December, 2016, retrospectively registered).

## Background

Children with cerebral palsy (CP) have disabilities both from motor impairment and from related disorders in other functions, including sensation, cognition, communication, vision, and behavior. Although various strategies have been used to improve neurologic impairment in patients with CP, most strategies used to date are complementary therapies, and there is currently no medical treatment that can repair the damaged nervous tissues [1]. Recent studies revealed that persistent neuroinflammation and associated apoptosis in brains affected by CP could be therapeutic targets [2]. Apoptosis is an attractive target because anti-apoptotic agents could be used to reverse apoptosis during a therapeutic time window after hypoxia-induced injury [3]. Other potential therapeutic targets include hematopoietic growth factors, such as erythropoietin (EPO) and granulocyte colony-stimulating factor (G-CSF), which influence the proliferation of neural stem and progenitor cells. EPO and G-CSF have specific receptors in the brain and both factors are produced in the brain [4, 5]. Therefore, EPO and G-CSF have been investigated for their ability to stop neurodegenerative conditions [6, 7].

Recently, cell therapy has emerged as a potential treatment for patients with CP. Bone marrow (BM)- or cord blood (CB)-derived mesenchymal stem cells (MSCs) have generally been used for cell therapy. In addition, intravenous CB mononuclear cells (CB MNCs) or intrathecal BM MNCs have been also assessed for safety and efficacy in children with CP [8–12]. Studies show that in children with CP recovery from neurologic impairment is promising, but not complete, following stem cell therapy with CB MNCs. However, there are limitations to the use of CB MNCs and BM MNCs for repeated therapy, as there is only one opportunity to collect CB MNCs, and the procedure for extracting BM MNCs is very invasive in children with CP.

We hypothesized that mobilized peripheral blood mononuclear cells (mPBMCs) would be a better source of cell therapy for children with CP, if these cells had a similar neuroregenerative potential to BM/CB MNCs. Multipotent precursor cells exist in peripheral blood, and a fraction of elutriated blood cells from normal individuals contains MNCs that have the potential to be MSCs [13]. There are several advantages to using mPBMCs for cell therapy in children with CP: the G-CSF that is used to mobilize peripheral blood mononuclear cells (PBMCs) has neuroregenerative potential; the collection and fractionation of stem cells can be repeated; and, the therapy is suitable for most children with CP. So far, there have been no clinical trials of cell therapy with autologous mPBMCs for children with CP. The current study describes a randomized, double-blind, cross-over study of intravenous G-CSF followed by infusion with autologous mPBMCs in children with CP to determine the safety and feasibility of the procedure, as well as the potential efficacy for improving neurological impairment.

## Methods

### Study design

**Fig. 1:**
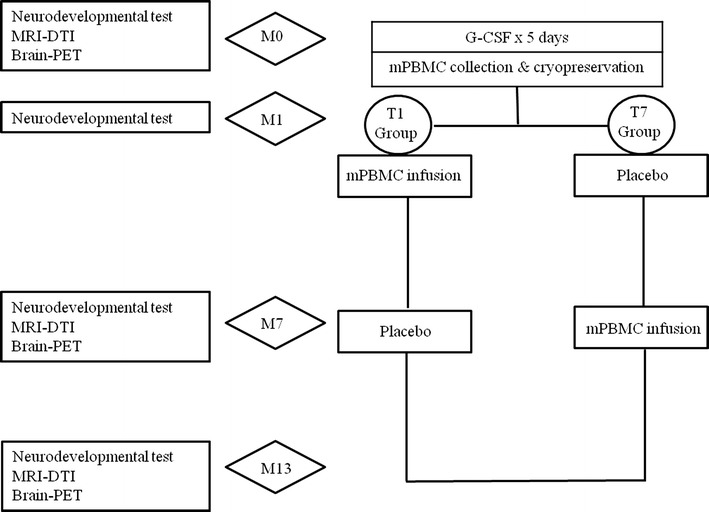
Design of the clinical study. *MRI-DTI* magnetic resonance imaging-diffusion tensor imaging; *PET* positron emission tomography; *G-CSF* granulocyte colony-stimulating factor; *mPBMC* mobilized peripheral blood mononuclear cell. M0, M1, M7 and M13 refer to months after enrollment. T1 and T7 refer to a group who received mPBMC at 1 and 7 months of study, respectively

Patients were included in the study if they were between 2 and 10 years of age and had a non-severe type of CP After baseline studies at enrollment (M0), intravenous G-CSF of 10 μg/kg was administered for 5 days. On the fifth day mPBMCs were collected with a single-day course of apheresis and then cryopreserved (Fig. 1). The detailed apheresis procedure is described in our previous study [14]. One month after cryopreservation of the mPBMCs (M1), patients were randomized to receive either mPBMCs or placebo. Six months after randomization (M7), cross-over infusion of mPBMCs or placebo was performed and the patients were observed for another 6 months. During the study, comprehensive physiotherapy and occupational therapy for individual patients were not modified. We assessed the neurodevelopmental status of patients at M0, M1, M7, and M13 after enrollment using various evaluation tools for neurodevelopmental tests. Brain magnetic resonance imaging-diffusion tensor image (MRI-DTI) and brain positron emission tomography-computed tomography (PET-CT) were used to evaluate the anatomical and functional changes in the brain at enrollment (M0), M7, and M13. This study was approved by the Institutional Review Board of Hanyang University Hospital (201103002).

### Evaluation of neurodevelopment

We assessed the parents’ feeling for the changes of motor or cognitive functions during study periods of their children, which even could not provide the clear and objective information on the neurodevelopmental evaluation. Comprehensive neurodevelopmental examinations were performed using the Denver development screening test II (DDST-II) to assess gross developmental screening, the pediatric evaluation of disability inventory (PEDI) to assess detailed developmental, the gross motor function classification system (GMFCS) to assess gross motor function staging, the gross motor function measure-88 (GMFM) to assess detailed motor function, the manual ability classification system (MACS) to assess fine motor staging, and the quality of upper extremity skill test (QUEST) to assess fine motor function. The results for each examination tool were evaluated by well-trained physical and occupational therapists, and therapeutic responses were comprehensively assessed by rehabilitation specialists.

### Neuroimaging studies

#### Brain MRI

All patients underwent MRI examination using a 3.0T system (Achieva, Philips, Best, Netherlands). Conventional images including axial T1-weighted, T2-weighted, and fluid-attenuated inversion recovery (FLAIR) were obtained for the anatomical evaluation. DTI data were obtained for the functional evaluation using a single shot echo planar sequence with the following parameters; 15 diffusion gradient directions, maximum b value = 800 s/mm^2^, TR/TE = 9000/55 ms, slice thickness = 2 mm. The DTI datasets were transferred to a workstation for processing. Fractional anisotropy (FA) and apparent diffusion coefficient (ADC) values for 18 regions of interest (ROIs) were obtained from the DTI data. All ROIs were set and analyzed by a pediatric neurologist.

#### Brain PET-CT scanning

Brain PET images were acquired using a dedicated PET-CT system (Biograph 6, Siemens Medical System, Knoxville, TN) at M0, M7, and M13 to monitor metabolic improvements of the brain. The patients fasted at least 6 h prior to PET-CT scanning. After intravenous injection of ^18^F-FDG (3.7 MBq/kg, 33–207 MBq), patients waited for 60 min in a dark room with a dim light before imaging, while ^18^F-FDG was distributed in brain. PET scans were obtained for 10 min, and images were reconstructed with a 168 × 168 matrix (pixel size = 1.95 × 1.95 mm with a slice thickness of 3.0 mm), and the ordered subset expectation maximum iterative reconstruction algorithm, an 5 mm Gaussian filter, and a 30 cm field of view. Two board-certified nuclear medicine physicians reviewed all three set of individual subject by visual assessment in consensus to check for the differences between the mPBMC group and placebo group, as well as T1 group and T7 group.

### Data analyses

We analyzed the following: safety and yield of G-CSF-mobilized PBMC collection; outcomes at M13; the booster effect of mPBMC infusion (by comparing the mPBMC group and the placebo group); and, outcomes according to mPBMC infusion time (T1 group: mPBMC infusion at 1 month after G-CSF infusion; T7 group: mPBMC infusion at 7 months after G-CSF infusion). The statistical software SAS for Windows (version 9.4, Cary, NC, USA) was used for data analysis. Logistic regression was used to analyze parental assessments, and the Mann–Whitney *U* test was used to analyze the neurodevelopment tests. The Wilcoxon rank sum test was used to analyze data from the brain MRI-DTI and PET-CT scans. P values ≤0.05 were considered to be statistically significant.

## Results

### Demographic data

Fifty-seven patients were enrolled in the current study and their mean age and body weight were 4.3 ± 1.9 years (range 2–10 years) and 16.6 ± 4.9 kg (range 11.6–56.0 kg), respectively. Types of CP were as follows: 31 diplegia, 15 hemiplegia, 11 others (triplegia, ataxic, athetoid). Possible causes of CP were periventricular leukomalacia (n = 37), hypoxic ischemic encephalopathy (n = 2), intracranial hemorrhage (n = 2), and unknown (n = 16). Forty-seven patients for whom complete study data were available were included in this analysis.

### Safety of G-CSF administration and yield of PBMCs

G-CSF-mobilized PBMC collection was safe in children with CP. We observed only two cases of fever and one episode of irritability during the G-CSF infusion. Transient hemoglobinuria (n = 3) and abdominal pain (n = 1) were reported during the mPBMC infusion, and these were resolved with supportive treatments. The total nucleated cell (TNC) count of mPBMCs was 5.97 ± 1.99 × 10^8^/kg, and the TNC count of CD34^+^ cells was 3.07 ± 2.1 × 10^6^/kg. Pre-freezing and post-thawing cell data were not significantly different between the two groups (T1 and T7), except for the post-thawing TNC counts, where the TNC count was significantly higher in the T7 group (Table 1).

**Table 1:** Cell counts of cryopreserved and infused mPBMCs

		T1 (N = 28)	T7 (N = 29)	*p*
Pre-freezing	TNC (×10^8^/kg)	5.84 ± 2.18	6.09 ± 1.89	0.574
	CD34 (×10^6^/kg)	3.16 ± 2.72	2.99 ± 1.40	0.678
Post-thawing	TNC (×10^8^/kg)	4.63 ± 2.88	6.20 ± 1.94	0.018
	CD34 (×10^6^/kg)	1.92 ± 1.99	1.75 ± 1.07	0.837

*mPBMC* mobilized peripheral blood mononuclear cell; *TNC* total nucleated cell; T1 and T7 refer to a group who received mPBMC at 1 and 7 months of study, respectively

### Parental assessments

Even before randomization for mPBMC infusion at M1 (i.e. during the one month after G-CSF administration), functional changes were noted by parents of 27 patients from the total group of 47 patients. After randomization, at M13, functional changes were noted by parents of 41 patients in the mPBMC group and by parents of 45 patients in the placebo group, although this difference was not statistically significant. There were also no significant differences in the functional changes recorded by parents at M13 between the T1 and T7 groups (Table 2).

**Table 2:** Number of patients for functional improvement by parental assessment according to randomization (before vs after) and time of mPBMC infusion (T1 vs T7)

	Before randomization	After randomization			T1	T7	p*
		Placebo	mPBMC	*p* ^+^			
Cognitive function	19	31	23	0.09	8	11	0.59
Motor function	3	0	2	0.15	0	0	–
Cognitive + motor function	5	14	16	0.65	13	14	0.83
No interval change	20	2	6	0.13	1	0	0.28

*mPBMC* mobilized peripheral blood mononuclear cell; T1 and T7 refer to a group who received mPBMC at 1 and 7 months of study, respectively

^+^ *p* indicates statistical significances of items between placebo and mPBMC groups

*p indicates statistical significances of items between before T1 and T7 groups

### Neurodevelopmental tests

Although we observed no significant changes in the GMFCS and MACS domains, 20 of 47 patients (42.6%) showed overall improvement at M13 in the domains of GMFM, PEDI and QUEST. We defined overall improvement as a score change in GMFM >4 points and/or score changes in PEDI >7 points in at least three items. The clinical characteristics of patients according to their overall therapeutic response are shown in Table 3. There were no risk factors associated with therapeutic responses; however, the number of responders was significantly higher in the T7 group than in the T1 group (p = 0.028). When comparing T1 and T7 groups (Table 4), the only significant score improvements were in the overall GMFM and GMFM-crawling domains, and these improvements were observed between enrollment and completion of study (M0–M13). Furthermore, when we compared the change in score after randomization (M1–M13), the GMFM, GMFM-crawling and GMFM-sitting domains showed significant improvement in the T7 group compared to the T1 group. Although there were no significant differences in neurodevelopment score improvement between mPBMC and placebo groups after randomization, more significant score changes in the GMFM, PEDI, and QUEST domains were observed before randomization (Table 5).

**Table 3:** Clinical characteristics according to overall therapeutic response, as measured by neurodevelopment tests

	Total (number or mean ± SD)	Responder (N = 20)^a^	Non-responder (N = 27)	*p*
Sex				0.915
Male	25	11	14	
Female	22	9	13	
Age (years)	4.1 ± 1.9	3.2 ± 1.5	4.7 ± 1.9	0.968
Body weight (kg)	16.6 ± 7.70	13. 8 ± 2.57	18.5 ± 9.35	0.142
Infused TNC (×10^8^/kg)	5.2 ± 2.54	5.7 ± 1.52	4.8 ± 3.04	0.847
CP type				0.215
Diplegia	25	10	15	
Non-diplegia	22	10	12	
Infusion time				0.028
T1	22	5	17	
T7	25	15	10	

*CP* cerebral palsy; T1 and T7 refer to a group who received mobilized peripheral blood mononuclear cell at 1 and 7 months of study, respectively

^a^Responder refers to a patient showing overall improvement at M13 as measured by neurodevelopment tests

**Table 4:** Change in neurodevelopment test score according to time of mPBMC infusion

	M0–M13			M1–M13		
	T1	T7	*p*	T1	T7	*p*
GMFM	4.28	8.30	0.002	2.90	6.37	0.001
Lying	1.34	1.33	0.398	0.89	0.71	0.780
Sitting	2.65	9.08	0.103	1.74	7.35	0.044
Crawling	5.19	12.86	0.010	2.92	10.41	0.002
Standing	4.78	7.18	0.093	3.50	5.13	0.079
Walking	7.45	10.02	0.391	5.43	8.45	0.198
PEDI_selfcare	8.64	8.13	0.370	5.58	6.54	0.204
Mobility	8.86	10.00	0.856	6.35	6.53	0.621
Social function	13.78	9.33	0.327	9.54	6.28	0.296
Selfcare (caregiver)	7.88	10.92	0.228	5.54	8.44	0.455
Mobility (caregiver)	4.90	7.28	0.221	3.29	6.22	0.052
Social function (caregiver)	11.45	14.65	0.236	8.87	12.13	0.208
QUEST	21.28	25.66	0.394	13.79	18.44	0.183

*mPBMC* mobilized peripheral blood mononuclear cell; *GMFM* gross motor function measure; *PEDI* pediatric evaluation of disability inventory; *QUEST* quality of upper extremity skills test. M0, M1, and M13 refer to months after enrollment. T1 and T7 refer to a group who received mPBMC at 1 and 7 months of study, respectively

**Table 5:** Changes in neurodevelopment test scores according to randomization

	Before randomization (M0–M1)	After randomization (M1–M7 or M7–M13)			p*
		Placebo	mPBMC	*p* ^+^	
GMFM	1.6738	0.4000	0.3725	0.479	0.001
PEDI_C	2.2787	0.5180	0.4928	0.764	0.006
PEDI_M	3.0212	0.6187	0.5419	0.998	0.005
PEDI_F	3.6106	0.6665	0.6536	0.930	0.042
PEDI_CC	2.4127	0.6115	0.5549	0.736	0.057
PEDI_MC	1.3191	0.5366	0.2558	0.092	0.030
PEDI_FC	2.5148	1.1481	0.6079	0.040	0.022
QUEST	9.0497	1.0815	1.3392	0.949	0.047

*mPBMC* mobilized peripheral blood marrow cell; *GMFM* gross motor function measure; *PEDI_C* pediatric evaluation of disability inventory self-care; *PEDI_M* pediatric evaluation of disability inventory mobility; *PEDI_F* pediatric evaluation of disability inventory social function; *PEDI_CC* pediatric evaluation of disability inventory mobility self-care with caregiver assistance; *PEDI_MC* pediatric evaluation of disability inventory mobility with caregiver assistance; *PEDI_FC* pediatric evaluation of disability inventory mobility social function with caregiver assistance; *QUEST* quality of upper extremity skills test

M0, M1, M7 and M13 refer to months after enrollment

^+^ *p* indicates statistical significances of items between placebo and mPBMC groups

* *p* indicates statistical significances of items between before randomization and after randomization

### Brain MRI-DTI scanning

In the MRI-DTI scans, there was a trend of increasing FA values and decreasing ADC values over time (M0–M13). However, these trends were not statistically significant, and there were also no significant differences between mPBMC and placebo groups (M1–M7 and M7–M13) in FA or ADC values in any ROIs. To evaluate the effect of mPBMC infusion on the changes in MRI-DTI scans, we analyzed the data according to the time of mPBMC infusion. The FA and ADC values were significantly greater in the T1 group than in the T7 group for the left corona radiata (CRL), left posterior limb of internal capsule (PLL), and the ADC values were significantly decreased in genu ROIs between M0 and M13 (Table 6), and in PLL ROI between M0 and M7 (Table 6). However, there were no significant differences in any ROI between M7 and M13.

**Table 6:** Changes in FA and ADC values

ROI	FA			ADC		
	T1	T7	*p*	T1	T7	*p*
*a Changes in FA and ADC values between M0 and M13*						
CRR	0.028	0.035	0.849	0.017	−0.018	0.723
CRL	0.056	0.029	0.032	−0.016	−0.015	0.935
PLR	0.057	0.020	0.144	−0.023	−0.013	0.643
PLL	0.039	0.018	0.010	−0.057	0.039	0.010
MBR	−0.010	0.028	0.397	0.001	0.121	0.723
MBL	0.024	0.001	0.349	−0.079	0.004	0.238
PonsR	−0.010	0.011	0.071	−0.047	−0.051	0.849
PonsL	0.027	0.035	0.892	−0.049	0.016	0.041
PTRR	0.023	0.010	0.643	0.008	0.022	0.683
PTRL	0.016	−0.013	0.367	−0.003	−0.009	0.935
FR	0.023	0.006	0.807	−0.003	0.008	0.978
FL	−0.003	0.000	0.605	−0.023	0.028	0.238
TR	0.046	0.004	0.367	−0.154	−0.045	0.683
TL	0.043	0.030	0.683	−0.002	0.003	0.849
OR	0.024	0.023	0.935	0.033	0.009	0.531
OL	0.023	0.039	0.461	0.025	−0.035	0.605
Genu	0.055	0.027	0.238	−0.35	−0.001	0.026
Spl	0.019	0.044	0.285	0.004	−0.112	0.091
*b Changes in FA and ADC values between M0 and M7*						
CRR	−0.011	0.041	0.070	−0.055	−0.034	0.845
CRL	0.017	0.009	0.499	−0.059	−0.033	1.000
PLR	0.003	−0.005	0.963	−0.058	−0.019	0.767
PLL	0.028	0.016	0.521	−0.084	0.009	0.020
MBR	−0.008	−0.003	0.521	−0.023	0.180	0.181
MBL	0.017	−0.007	0.481	−0.028	−0.053	0.913
PonsR	0.006	−0.007	0.964	−0.074	−0.017	0.105
PonsL	0.028	0.011	0.775	−0.063	−0.003	0.284
PTRR	−0.011	0.019	0.424	−0.033	0.019	0.839
PTRL	−0.003	−0.011	0.696	−0.045	−0.071	0.462
FR	0.013	−0.015	0.189	−0.063	−0.002	0.313
FL	−0.003	−0.035	0.298	−0.060	0.011	0.599
TR	0.006	0.007	0.845	−0.125	−0.031	0.940
TL	0.007	0.017	0.408	−0.047	−0.011	0.775
OR	−0.010	0.002	0.397	−0.044	−0.012	0.415
OL	−0.005	0.005	0.754	−0.095	−0.048	0.397
Genu	0.032	0.002	0.271	−0.107	0.025	0.070
Spl	−0.026	0.001	0.093	0.033	−0.040	0.490

M0, M7, and M13 refer to months after enrollment

*FA* fractional anisotropy; *ADC* apparent diffusion coefficient; *ROI* region of interest; *CRR* corona radiata, right; *CRL* corona radiata, left; *PLR* internal capsule, posterior limb, right; *PLL* internal capsule, posterior limb, left; *MBR* midbrain, right; *MBL* midbrain, left; *PonsR* pons, right; *PonsL* pons, left; *PTRR* posterior thalamic radiation, right; *PTRL* posterior thalamic radiation, left; *FR* frontal, right; *FL* frontal, left; *TR* temporal right; *TL* temporal left; *OR* occipital right; *OL* occipital left; *Spl* splenium

### Brain PET-CT scanning

Although we observed metabolic changes to the cerebellum, thalamus and cerebral cortex in the brain PET-CT, there were no significant differences in the incidence of metabolic changes between the mPBMC and placebo groups, or between the T1 and T7 groups.

## Discussion

We have performed a clinical trial using G-CSF and mobilized PBMCs in patients with CP, based upon the following backgrounds. The use of G-CSF in children with CP is ethical and beneficial because it is already proven to be safe in normal volunteer donors [15, 16], and G-CSF has the potential to induce neuroregeneration in patients with neurodegenerative diseases [5, 7]. Also, the mobilization and apheresis of mPBMCs in children are safe and effective [15], and mPBMCs contain MSCs which can be isolated and then secrete various cytokines potentially able to repair the damaged tissues [13, 17]. Additionally, we have previously reported upon the safety of administering G-CSF and collecting mPBMCs in children with CP [14], and we have also reported on the intracellular expression of neurotrophic factors and inflammatory cytokines, which could exert a neuroregenerative effect, in mPBMCs from children with CP [17].

In the current study, we demonstrated that G-CSF administration and collection/reinfusion of mPBMCs were safe and tolerable in children with CP. A single-day apheresis in children with CP yielded TNC counts of 5.97 ± 1.99 × 10^8^/kg, and CD34^+^ cell counts of 3.07 ± 2.1 × 10^6^/kg. These numbers are sufficient for hematopoietic reconstitution, although the target dose of MNCs or MSCs for cell therapy is yet to be determined. The yield of mPBMCs from children with CP was comparable to the yield from normal pediatric donors and patients with nonhematologic disease, reported in other studies [18, 19]. We cryopreserved all collected mPBMCs for at least 1 month and then reinfused them (after randomization) at 1 month or 7 months. With this protocol, we tried to reveal the neuroregenerative effect of G-CSF alone, without circulating mPBMCs, as well as any possible augmented effect following reinfusion with mPBMCs.

We observed that 42.6% of patients showed overall responses in the neurodevelopment tests than would normally be expected. In addition, the largest neurodevelopment score improvements were obtained during 1 month after G-CSF administration followed by mPBMC collection (M0–M1), suggesting that G-CSF alone, irrespective of circulating mPBMCs, can counter neurological impairment in children with CP. We also tried to assess the booster effects of mPBMCs on neurodevelopmental functions. Compared to the placebo group, the group that had a mPBMC infusion at 7 months experienced a greater improvement in neurodevelopmental functions than the group receiving a mPBMC infusion at 1 month. However, it should be pointed out that the higher infused cell doses in the T7 group were associated with neurodevelopmental improvement, because there is, as yet, no evidence that cell dose has an effect on neurologic improvements. In addition, Hara [20] and Hayashiji [21] reported that G-CSF positively affects the recovery of muscle mass, therefore delayed effect of G-CSF on muscle regeneration may be contributed to synergistic improvement of neurodevelopmental functions in T7 group. Contrary to the results from neurodevelopment tests, MRI-DTI showed greater increase of FA values in the CRL, PLL, and Genu ROIs in the T1 group than in the T7 group, indicating that the neuroregenerative effect of G-CSF coupled with mPBMC infusion is higher at 1 month than at 7 months.

Given these differences between the results of neurodevelopment tests and the changes observed in MRI-DTI scans, the neuroregenerative effect of mPBMC reinfusion may be minimal and/or the additive effect of G-CSF on the central nervous system as well as muscles could be considered. Furthermore, the collection and reinfusion of mPBMCs after G-CSF administration may not be essential because G-CSF could synergize with endogenous circulating mPBMCs. Although we could not demonstrate the effect of G-CSF on muscle and an additional effect of mPBMCs on the changes of FA/ADC values in the MRI-DTI scans, several investigators have found a correlation between ROI-based FA and clinical motor outcome in children with CP [22–24]. Therefore, future studies using MRI-DTI would be needed to reveal the association of infusion timing or cell doses of mPBMCs and clinical outcomes in children with CP.

## Conclusion

We observed a neuroregenerative potential of intravenous G-CSF followed by mPBMC reinfusion. The neurodevelopmental improvement observed may have been caused by G-CSF alone without a contribution from the reinfused mPBMCs. Further studies using higher concentrations of mPBMCs which could be collected by repeated cycles of apheresis or using repeated cycles of G-CSF alone are needed to clarify any benefit of mPBMC reinfusion or G-CSF alone.

## Abbreviations

G-CSF: granulocyte colony-stimulating factor
mPBMC: mobilized peripheral blood mononuclear cells
CP: cerebral palsy
M0: month at enrollment
M1: 1 month after enrollment
M7: 7 months after enrollment
M13: 13 months after enrollment
MRI-DTI: magnetic resonance imaging-diffusion tensor imaging
FDG: ^18^F-fluorodeoxyglucose
PET-CT: positron emission tomography-computed tomography
T1: group for mPBMC infusion at 1 month
T7: group for mPBMC infusion at 7 months
EPO: erythropoietin
BM: bone marrow
CB: cord blood
MSCs: mesenchymal stem cells
MNCs: mononuclear cells
DDST II: Denver development screening test II
PEDI: pediatric evaluation of disability inventory
GMFCS: gross motor function classification system
MACS: manual ability classification system
QUEST: quality of upper extremity skill test
FLAIR: fluid-attenuated inversion recovery
FA: fractional anisotropy
ADC: apparent diffusion coefficient
ROIs: regions of interest
TNC: total nucleated cell
CRL: left corona radiata
PLL: left posterior limb of internal capsule
